# Further studies on the diversity of *Cylicospirura* Vevers, 1922 (Nematoda: Spirocercidae) in African carnivores, with description of a new species

**DOI:** 10.1017/S0031182024000659

**Published:** 2024-06

**Authors:** Kerstin Junker, Lin-Mari de Klerk-Lorist, Chris Foggin, Coralie Martin, Yasen Mutafchiev

**Affiliations:** 1 National Collection of Animal Helminths, Epidemiology, Parasites and Vectors Programme, Agricultural Research Council-Onderstepoort Veterinary Institute, Private Bag X05, Onderstepoort 0110, South Africa; 2 Department of Agriculture, Land Reform & Rural Development, State Veterinary Office and Laboratory, Kruger National Park, PO Box X12, Skukuza 1350, South Africa; 3 Victoria Falls Wildlife Trust, PO Box 159, Victoria Falls, Zimbabwe; 4 UMR7245, MCAM, Museum National d'Histoire Naturelle, Paris, 75005, France; 5 Department of Animal Diversity and Resources, Institute of Biodiversity and Ecosystem Research, Bulgarian Academy of Sciences, Sofia, Bulgaria

**Keywords:** Africa, *Crocuta crocuta*, *Cylicospirura*, *Felis lybica*, *Hyaena hyaena*, morphometrics, *Panthera pardus*, Spirurida, taxonomy

## Abstract

*Cylicospirura* Vevers, 1922 is a genus of gastrointestinal spirurid nematodes parasitizing mainly felid but also canid, hyaenid and dasyurid hosts. Presently, 11 species are recognized worldwide, of which 4, *Cylicospirura subaequalis*, *Cylicospirura felinea*, *Cylicospirura crocutae* and *Cylicospirura pardalis*, have been recorded from African carnivores. In the present study, we describe *Cylicospirura phiri* n. sp. from hyaenas, namely *Crocuta crocuta* (type host) in Zimbabwe and *Hyaena hyaena* in Cameroon. The new species is the second species in the genus with bicuspid teeth. Furthermore, it can be distinguished from its congeners by a combination of characters, such as the absence of accessory teeth, the length and shape of the muscular oesophagus, position of the nerve ring, deirids and excretory pore in relation to the muscular oesophagus, the position of the vulva, spicule length and the shape of the female tail. Additionally, based on new material, detailed morphological descriptions are provided for *C. crocutae* and *C. pardalis* whose original descriptions were based on fragmented material. The material from *Felis lybica*, currently deposited as *C. subaequalis*, is described as *C. felinea*. First-time scanning electron micrographs are presented for the 4 species confirmed in African carnivores.

## Graphical abstract

**Figure:**
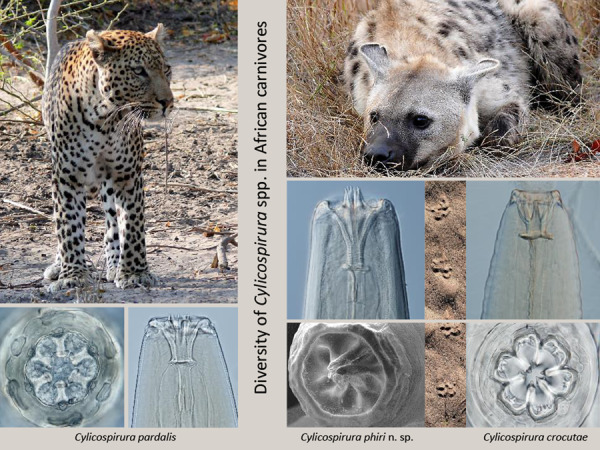

## Introduction

Established by Vevers (1922) to accommodate the single species *Cylicospirura subaequalis* (Molin, 1860) (syn. *Spiroptera subaequalis* Molin, 1860), the genus *Cylicospirura* Vevers, 1922 presently comprises 11 species from the gastrointestinal tract of mainly felid but also canid, hyaenid and dasyurid hosts (Junker *et al*., 2013).

To date, 4 species of *Cylicospirura* have been recorded from African carnivores, 1 species, *Cylicospirura felinea* (Chandler, 1925) from *Felis lybica* Forster and *Vulpes vulpes* Linnaeus in Algeria (Seurat, 1913, 1915), as well as from a domestic cat in South Africa (Junker *et al*., 2006), and 3 species from large carnivores. *Cylicospirura subaequalis* was listed from *Crocuta crocuta* (Erxleben) in Kenya (Round, 1968) and Ethiopia (Graber *et al*., 1980), from *Panthera leo* (Linnaeus) in the Democratic Republic of the Congo (Sandground, 1930) and the Central African Republic (Graber and Thal, 1980), as well as from *Panthera pardus* (Linnaeus) in Sierra Leone (Round, 1968). *Cylicospirura crocutae* Junker and Mutafchiev, 2013 was described from *Cr. crocuta* in Zimbabwe and *Cylicospirura pardalis* Junker and Mutafchiev, 2013 from *P. pardus* in South Africa (Junker *et al*., 2013). Junker *et al*. (2013), prompted by the discovery of the 2 new species of *Cylicospirura* in *Cr. crocuta* and *P. pardus*, cautioned that earlier listings of *C. subaequalis* in large African carnivores might require further studies, especially in view of the fact that many previous records had not been accompanied by morphological descriptions.

As pointed out by Vevers (1922), specimens from *F. lybica* (as *Felis ocreata* Gmelin) in Algeria, assigned to *Spirocerca subaequalis* by Seurat (1913), differed from previous descriptions of this species by the presence of tricuspid teeth. These are clearly illustrated by Seurat (1913) as well as mentioned in his detailed description of the specimens. Consequently, Sandground (1930) referred Seurat's (1913) specimens to *C. felinea*. Specimens from a domestic cat in South Africa, examined by Junker *et al*. (2006), corresponded well to the illustrations of Seurat (1913) as well as to the redescription of *C. felinea* by Pence *et al*. (1978) and were thus also assigned to *C. felinea*. Despite these earlier taxonomic works on *Cylicospirura* species (Seurat, 1913; Vevers, 1922; Sandground, 1930; Pence *et al*., 1978; Junker *et al*., 2013), the diversity of this interesting genus in Africa remains insufficiently studied.

Recently, new material collected during necropsies of *Cr. crocuta* in Zimbabwe and *P. pardus* in South Africa allowed us to further investigate the diversity of *Cylicospirura* in large African carnivores. As a result, we are able to add morphological data to the original descriptions of *C. crocutae* and *C. pardalis,* which had been based on highly fragmented material (Junker *et al*., 2013). The same *Cr. crocuta* from Zimbabwe harboured a second species of *Cylicospirura*, which is described here as a new species. Further, we report the results of the re-examination of some of the collection material deposited in the Muséum National d'Histoire Naturelle, Paris, France (MNHN) and present the first scanning electron microscopy studies of the morphology of species of *Cylicospirura*.

## Materials and methods

An adult male spotted hyaena, *Cr. crocuta*, that had been run over by a car in the vicinity of Victoria Falls, Zimbabwe (-18.01702, 25.8359) was necropsied on 17 July 2017 on behalf of the Parks and Wildlife Management Authority, Zambezi National Park. Nematodes were found free in the cranial oesophagus as well as in open granulomata in the stomach.

In South Africa, an adult male leopard had to be euthanized in close proximity to the Lower Sabie Rest Camp (-25.12270, 31.91574), Kruger National Park, on 07 April 2019 (Management Biodiversity Act: TOPS permit number 07620, see Junker and de Klerk-Lorist (2020) for additional details; and Department of Environmental Affairs Standing Permit number 07612). During necropsy, nematodes were collected from granulomata in the stomach (pyloric outlet), duodenum and jejunum.

The following specimen lots from the MNHN were examined: MNHM IN YT112 and MNHN IN YT113.

Helminths were fixed and stored in 70% ethanol and cleared in lactophenol for morphological examination. Apical views were cut using a razor blade. Specimens were studied under a compound microscope (Olympus BX51) equipped with a drawing tube and digital camera (Olympus DP72). Photos and measurements were taken with the aid of digital imaging software (Olympus cellSens Dimension, version 1.4.1). Specimens used for SEM were dehydrated through a graded ethanol series, immersed in hexamethyldisilazane for 20 min, air-dried, coated with gold–palladium in an Emitech K500X sputter coater and examined using a Tescan LYRA 3 XMU FEG/SEMxFIB microscope at an accelerating voltage of 10 kV. All measurements are given in micrometres unless otherwise indicated; the range is followed by the mean in parentheses, if justified by the number of specimens. If the number of specimens in which a given feature could be measured differed from the total number of specimens examined, this number (*n*) is provided in parentheses. Newly collected specimens were deposited in the National Collection of Animal Helminths, ARC-Onderstepoort Veterinary Institute, South Africa (NCAH) and in the collection Evertebrata Varia at the Naturhistorisches Museum Wien (NHMW). Nomenclature of Felidae follows Kitchener *et al*. (2017), that of the remaining hosts Wilson and Reeder (2005).

## Results

### Order: Spirurida Chitwood, 1933 Superfamily: Spiruroidea Oerley, 1885 Family: Spirocercidae Chitwood and Wehr, 1932 Genus: *Cylicospirura* Vevers, 1922 *Cylicospirura phiri* Junker and Mutafchiev n. sp.

*Type host*: *Crocuta crocuta* (Erxleben) (Carnivora: Hyaenidae); spotted hyaena.

*Other host*: *Hyaena hyaena* (Linnaeus) (Carnivora: Hyaenidae); striped hyaena.

*Type locality*: Vicinity of Victoria Falls, Zimbabwe (17.vii.2017; -18.0170, 25.8359).

*Site in host*: Free, in cranial part of oesophagus (5 males, 13 females). Two males in open granulomata in stomach.

*Intensity of infection*: A single spotted hyaena harboured 20 specimens.

*Type material*: ex *Crocuta crocuta*: NCAH/2024/004 (holotype male from the oesophagus), NCAH/2024/005 (paratypes: 3 males, 8 females, 1 female posterior from the oesophagus); NCAH/2024/006 (1 male paratype from the stomach). NHMW-ZOO-EV-A-21540 (paratypes: 1 male, 2 females), NHMW-ZOO-EV-M-5895 (fragments of 1 male and 1 female).

*Additional material*: ex *Hyaena hyaena* from Cameroon (26.ii.1971), collected by J Thal; 1 male and 4 gravid females (MNHM IN YT112); site of infection, prevalence and intensity of infection not specified.

*ZooBank registration*: urn:lsid:zoobank.org:act:C942BAAD-0B77-406E-9F9B-198A77C7609D.

*Etymology*: The species name, a noun in apposition, refers to the hosts of this parasite. ‘Phiri’, pronounced [pi:ri:], is the Setswana word for hyaena (le Roux, 1991).

#### Description (Figs 1–4; Tables 1 and 2)

**Figure 1.**
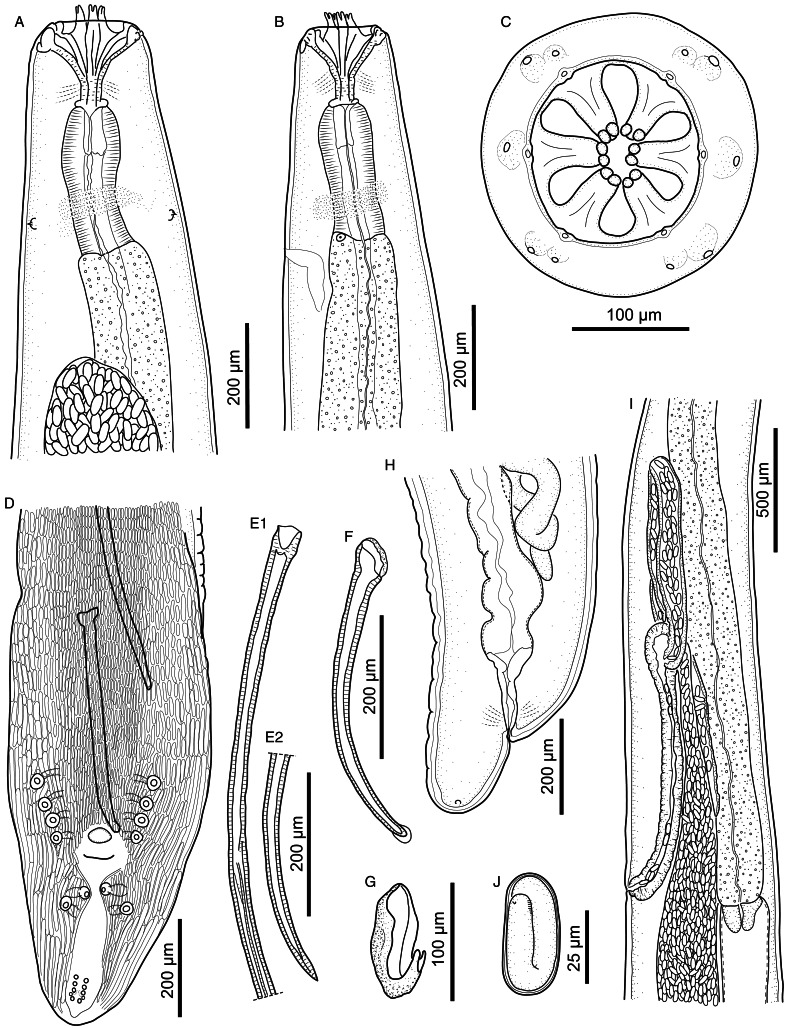
*Cylicospirura phiri* n. sp. ex *Crocuta crocuta.* (A) Anterior end, female, ventral view; (B) Anterior end, male, lateral view; (C) Anterior extremity, female, apical view; (D) Posterior end, male, ventral view; (E) Left spicule, proximal end (E1) and distal end (E2), lateral view; (F) Right spicule, lateral view; (G) Gubernaculum, lateral view; (H) Posterior end female, lateral view; (I) Oesophago-intestinal junction and terminal part of female genital system, lateral view; (J) Egg.

**Figure 2.**
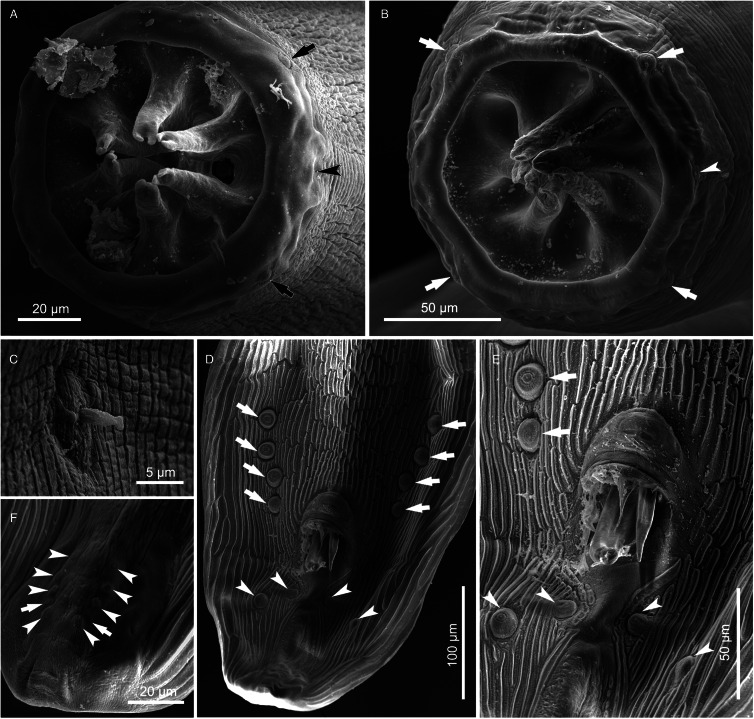
*Cylicospirura phiri* n. sp. ex *Crocuta crocuta*, SEM. (A) Male, apical view; (B) Female, apical view, note cephalic papillae (arrows) and one of the amphids (arrowhead); (C) Deirid, female; (D) Posterior extremity, male, note precloacal pairs of pedunculated papillae (arrows) and postcloacal pairs of pedunculated papillae (arrowheads); (E) Cloacal region, note precloacal pedunculated papillae (arrows) and postcloacal pairs of pedunculated papillae (arrowheads); (F) Tail extremity, male, note sessile papillae (arrowheads) and phasmids (arrows).

**Figure 3.**
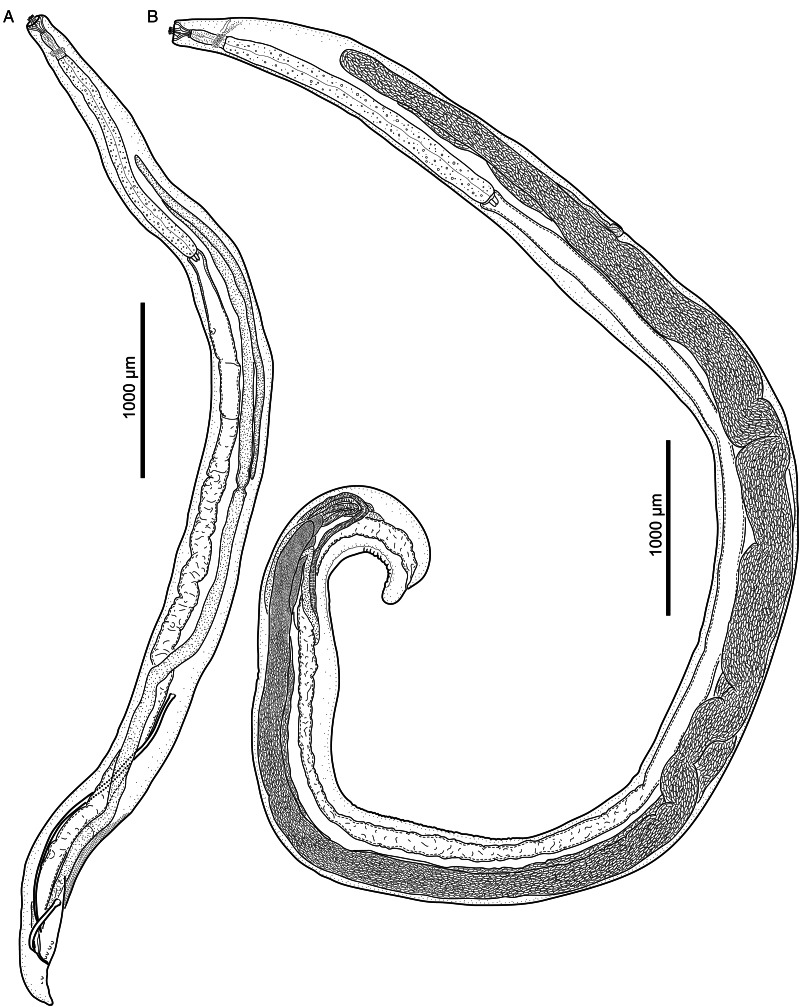
*Cylicospirura phiri* n. sp. ex *Hyaena hyaena.* (A) Male, habitus, lateral view; (B) Female, habitus, lateral view.

*General.* Medium-sized spirurid. Body cylindrical, bent dorsally (Fig. 3), tapering at both ends; maximum body width at oesophago-intestinal junction. Cuticle with delicate transverse striations, spaced approximately 2 *μ*m apart in males and 3 *μ*m apart in females. Mouth opening faintly hexagonal, with rounded edges (Fig. 2B). Head papillae composed of an inner circle of 6 small internal labial papillae in lateral and submedian positions, close to mouth opening, visible using light but not electron microscopy, and an outer circle of 4 external labial papillae and 4 larger cephalic papillae, adjacent to each other and aligned with base of submedian large teeth (Fig. 1C). Amphids aligned with base of lateral teeth. Buccal capsule large, heavily sclerotized with conspicuous transverse rugosities, funnel-shaped, with cylindrical base (Fig. 1A and B), armed with 6 large teeth, 2 lateral and 4 submedian, arising in posterior third of buccal capsule and projecting anteriorly beyond the mouth opening (Figs 1A and B and 2A); each tooth with 2 apical cusps with deep slit between them. Accessory teeth absent. Oesophagus divided into anterior muscular and posterior glandular part (Fig. 1A). Muscular oesophagus long and slender, a dilatation with valve close to its junction with buccal capsule; glandular oesophagus subtly increasing in width posteriorly, with widest part at oesophago-intestinal junction. Nerve-ring surrounding muscular oesophagus in posterior half; deirids minute, spine-like with flattened tip (Fig. 2C), just posterior to level of nerve ring (Fig. 1A and B); excretory pore slightly posterior to deirids (Fig. 1B).

**Figure 4.**
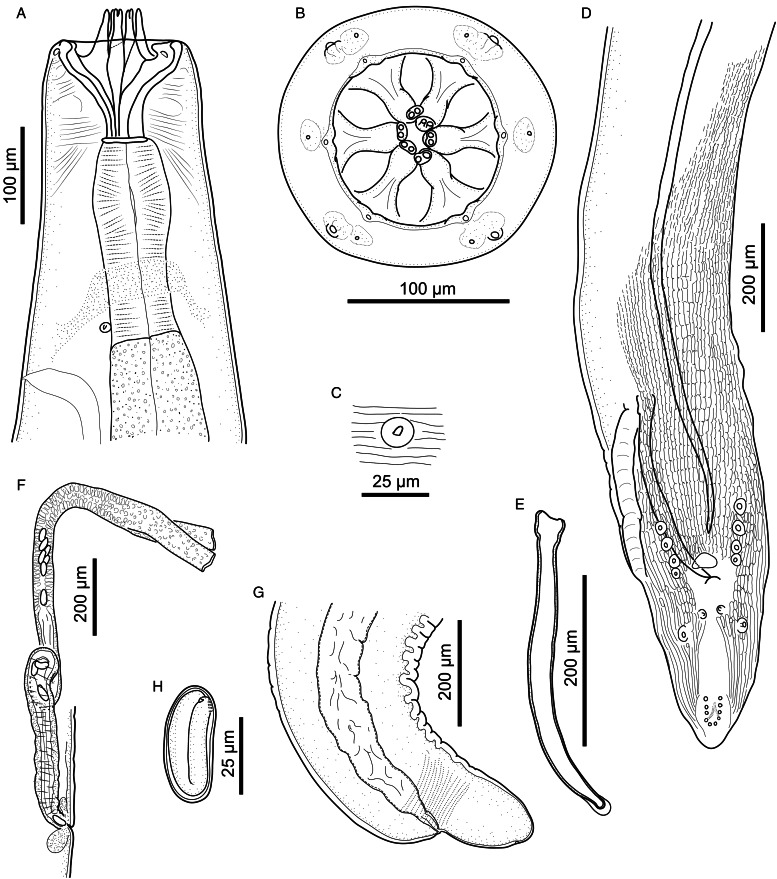
*Cylicospirura phiri* n. sp. ex *Hyaena hyaena.* (A) Anterior end, female, lateral view; (B) Anterior extremity, female, apical view; (C) Deirid; (D) Male, posterior end, ventral view; (E) Right spicule, lateral view; (F) Vagina, ovejector and distal part of uterus; (G) Posterior end, female, lateral view; (H) Egg.

**Table 1.** Morphometric data of *Cylicospirura* spp. males from African carnivores

Species	*C. crocutae*	*C. crocutae*	*C. pardalis*	*C. pardalis*	*C. phiri* n. sp.	*C. phiri* n. sp.	*C. felinea*
Source	Junker *et al*. (2013)	Present study	Junker *et al*. (2013)	Present study	Present study	Present study	Present study
Host species	*Crocuta crocuta*	*Crocuta crocuta*	*Panthera pardus*	*Panthera pardus*	*Crocuta crocuta*	*Hyaena hyaena*	*Felis silvestris ocreata*
Locality	Zimbabwe	Zimbabwe	South Africa	South Africa	Zimbabwe	Cameroon	Algeria
No. examined	*n* = 2	*n* = 5	*n* = 1^a^	*n* = 17	*n* = 7	*n* = 1	See text
Body length (mm)	22.7^b^	22.2–26.0^c^	–	18.5–32.2	11.5–13.3	6.5	20.8
Body width	480; 495	460–646	618	485–641	384–472	347	454
Buccal capsule length	139^b^	114–152	–	125–187	132–155	92	93–102
Buccal capsule, maximum width	131^b^	138–161	–	149–175	133–160	129	134–145
Muscular oesophagus length	592^b^	696–987	–	363–742^d^	239–276	159	474–564
Glandular oesophagus length (mm)	5.38^b^	4.94–8.63	3.10	2.72–4.52^d^	1.91–2.56	1.44	3.49–3.50
Total oesophagus length (mm)	5.97^b^	5.65–9.62	–	3.22–5.55^d^	2.19–2.83	1.60	3.96–4.06
Nerve ring from anterior end, distance	453^b^	382–524^c^	–	332–533^d^	304–343	196	316–377
Left spicule length (mm)	2.25; 2.24	2.22–2.55^c^	2.71^e^	2.49–2.86^d^	2.06–2.57	1.64	1.87–2.04
Right spicule length	564; 550	554–645^c^	568	517–701^d^	465–513	366	423–472
Gubernaculum length	144	80–90^c^	–	86–115^d^	57–93	64	–
Tail length	340; 325	310–355^c^	–	303–408	303–382	311	218–246

Measurements are in micrometres unless otherwise stated.

a Based on one anterior and one posterior fragment.

b Holotype only; otherwise holotype listed first.

c Based on four specimens.

d See text for number of specimens measured; TBL – total body length.

e Proximal part broken off.

**Table 2.** Morphometric data of *Cylicospirura* spp. females from African carnivores

Species	*C. crocutae*	*C. crocutae*	*C. pardalis*	*C. pardalis*	*C. phiri* n. sp.	*C. phiri* n. sp.	*C. felinea*
Source	Junker *et al*. (2013)	Present study	Junker *et al*. (2013)	Present study	Present study	Present study	Present study
Host	*Crocuta crocuta*	*Crocuta crocuta*	*Panthera pardus*	*Panthera pardus*	*Crocuta crocuta*	*Hyaena hyaena*	*Felis silvestris ocreata*
Locality	Zimbabwe	Zimbabwe	South Africa	South Africa	Zimbabwe	Cameroon	Algeria
No. examined	*n* = 2	*n* = 11	*n* = 1	*n* = 8	*n* = 10	*n* = 4	See text
Body length (mm)	–	33.4–36.9^a^	—	29.0–45.5	19.7–24.2	10.0–11.9	16.7–21.2
Maximum width	597; –	494–645	868	674–861	553–649	440–594	558–693
Buccal capsule length	162; 175	112–190	170	150–223	133–174	98–112	109–126
Buccal capsule, maximum width	137; 135	147–176	197	186–240	185–204^a^	154–168	140–160
Muscular oesophagus length	821; 814	757–1095^a^	498	665–869^a^	256–323	191–225	509–620
Glandular oesophagus length (mm)	6.03; 6.58	6.06–8.09^a^	3.5	3.87–4.99^a^	2.22–3.38	1.77–1.87	3.61–4.51
Total oesophagus length (mm)	6.85; 7.39	6.90–8.85	4.0	4.58–5.75^a^	2.50–3.71	1.99–2.09	4.12–5.13
Nerve ring from anterior end, distance	545; 568	458–588^a^	441	490–629^a^	332–361^a^	216–265	362–430
Vulva from anterior end, distance (mm)	3.2; 3.3	2.51–4.53^a^	3.4	4.11–5.51^a^	1.93–3.281	1.47–2.77	3.05–4.27
Tail length	–	90–149^a^	–	264–334	165–213	139–153	96–134
Egg size	32–35 × 17–20	34–37 × 18–23	35–38 × 14–16	36–38 × 14–17	38–41 × 16–18	37–39 × 16–18	39–40 × 20–22

Measurements are in micrometres unless otherwise stated.

a See text for number of specimens measured.

*Males* (based on 7 males from *Cr. crocuta*; except where otherwise indicated). Body 11.5–13.3 (12.7) mm long. Maximum body width 384–472 (418). Buccal capsule 132–155 (141) long, including cylindrical base 54–64 (58) long and 35–51 (39; *n* = 6) wide; maximum width of buccal capsule 133–160 (149). Teeth 103–123 (114; *n* = 4) long, measured from split from common base to tips, with slit between tips 11–14 (13; *n* = 6) deep. Nerve-ring, deirids and excretory pore at 304–343 (316), 338–424 (375) and 395–520 (445) from anterior extremity, respectively. Total length of oesophagus 2188–2826 (2397); i.e. 17.7–21.2% (18.9%) of body length. Muscular oesophagus 239–276 (258) long and 86–107 (99) wide at dilatation; glandular oesophagus 1912–2557 (2139) long and 166–214 (196) wide; ratio of muscular to glandular oesophagus length 1:6.9–9.5 (1:8.3). Testis extending just anterior to level of oesophago-intestinal junction in 4 males, and just posterior to it in 3 males; reflexion of testis at 2134–2942 (2552; *n* = 6) from anterior extremity. Tail coiled ventrally, 303–382 (348) long. Caudal alae narrow, 960–1348 (1167; *n* = 6) long. Area rugosa composed of distinct, parallel, longitudinal cuticular ridges; absent in area surrounding cloaca and subterminal papillae (Fig. 2D); extending 1663–2232 (2053) anteriorly from tip of tail. Cloaca slit-like, without conspicuous cuticular lips. Ten pairs of caudal papillae; pairs 1–4 ventrolateral, precloacal, pedunculated, approximately equidistant from each other and of similar size; pairs 5 and 6 postcloacal, pedunculated, pair 5 ventral, slightly anterior to ventrolateral pair 6 (Figs 1D and 2D and E); pairs 7–10 small, sessile, somewhat indistinct, arranged in subterminal group around cuticular pads near tip of tail; phasmids situated between pairs 9 and 10 (Fig. 2F). A single, large, sessile, median papilla anterior to cloaca present (Figs 1D and 2E). Spicules unequal and dissimilar; left spicule 2060–2569 (2446) long and slender, tapering to pointed tip (Fig. 1E); right spicule 465–513 (495) long, robust with rounded, hyaline distal tip (Fig. 1F); spicular ratio 1:4.2–5.3 (1:4.9). Gubernaculum present, irregularly shaped and weakly sclerotized, 57–93 (70; *n* = 3) long (Fig. 1G).

*Females* (based on 10 gravid females from *Cr. crocuta*; except where otherwise indicated). Body 19.7–24.2 (22.1) mm long. Maximum body width 553–649 (596). Buccal capsule 133–174 (155) long, including cylindrical base 41–61 (49; *n* = 9) long and 37–45 (41; *n* = 9) wide; maximum width of buccal capsule 185–204 (195; *n* = 9). Teeth 128–152 (140; *n* = 9) long, measured from split from common base to tips, with slit between tips 11–17 (13; *n* = 9) deep. Nerve-ring, deirids and excretory pore at 332–361 (352; *n* = 9), 359–447 (398; *n* = 9) and 452–559 (484; *n* = 9) from anterior extremity, respectively. Total length of oesophagus 2479–3707 (2955); i.e. 12.2–15.8% (13.4%) of body length. Muscular oesophagus 256–323 (286) long and 116–133 (124) wide at dilatation; glandular oesophagus 2222–3384 (2686) long and 186–219 (203) wide; ratio of muscular to glandular oesophagus length 1:8.5–10.7 (1:9.4). Uteri didelphic, opisthodelphic. Vulva without salient lips, opening at oesophago-intestinal junction (*n* = 6) or 152–720 (375; *n* = 4) anterior to it (Fig. 1I), i.e. at 9.7–14.0% (12.3%) of body length; vagina simple, directed anteriorly or posteriorly, 950–1243 (1098; *n* = 5) long, ovejector convoluted and largely obscured by eggs in uterine loops (Fig. 1I). Eggs in uterus thin-shelled with almost parallel sides and with well-developed first-stage larva (Fig. 1J); 38–41 (39; n = 10) × 16–18 (17; *n* = 10). Tail rounded (Fig. 1H), 165–213 (188) long; tail length to width at anus ratio 1:1.0–1.1 (*n* = 3). Phasmids at 20–33 (28) from tail tip.

Observations on material from *Hyaena hyaena* (Figs 3 and 4; Tables 1 and 2)

Despite being distinctly smaller in body size (Tables 1 and 2), the specimens from *H. hyaena* correspond well with the sample from *Cr. crocuta*. We found no meaningful discrepancies with respect to any important morphological characters, such as shape and arrangement of the bicuspid teeth, prominent cuticular ridges of the buccal capsule, position of the nerve-ring, deirids and excretory pore in relation to the muscular and glandular oesophagus or number and arrangement of head and caudal papillae or shape of the female tail. The main differences between the 2 sets of specimens are the shorter spicules in the males from *H. hyaena* compared to the males from *Cr. crocuta* and the varying position of the vulva in females. Moreover, the position of the excretory pore in specimens from *H. hyaena* is variable, and either on the level of the nerve-ring or posterior to it, on the level of the posterior part of the muscular oesophagus or just posterior to the junction between the muscular and glandular oesophagus (Fig. 4A).

#### Remarks

The shorter spicule length in the male from *H. hyaena* likely reflects the overall smaller body size of the parasite, which in turn might be the result of adaptation to a different host species or a geographic variation (Zapata and Jiménez, 2012), with *H. hyaena* from Cameroon in Central Africa and *Cr. crocuta* from Zimbabwe in the southern subregion of the continent. In females from *H. hyaena*, the vulva is posterior to the oesophago-intestinal junction in 3 specimens, but anterior to it in a single specimen, whereas in females from *Cr. crocuta*, the position of the vulva varied from opening at the oesophago-intestinal junction or anterior to it. Variation in the position of the vulva has also been reported in other Spirocercidae. In *Spirocerca lupi* (Rudolphi, 1819) from dogs in Vietnam, the position of the vulva ranged from at the oesophago-intestinal junction to well beyond it in gravid females (Hoa *et al*., 2021). Given the current data, we believe that these morphological discrepancies are within the intraspecific variability of the species.

In having a short, heavily sclerotized buccal capsule with 6 large teeth that arise from its posterior half (third), 6 pairs of pedunculated caudal papillae and a vulva positioned near the oesophago-intestinal junction, the present specimens from *Cr. crocuta* and *H. hyaena* conform to the generic diagnosis of *Cylicospirura* as presented by Junker *et al*. (2013).

**Figure 5.**
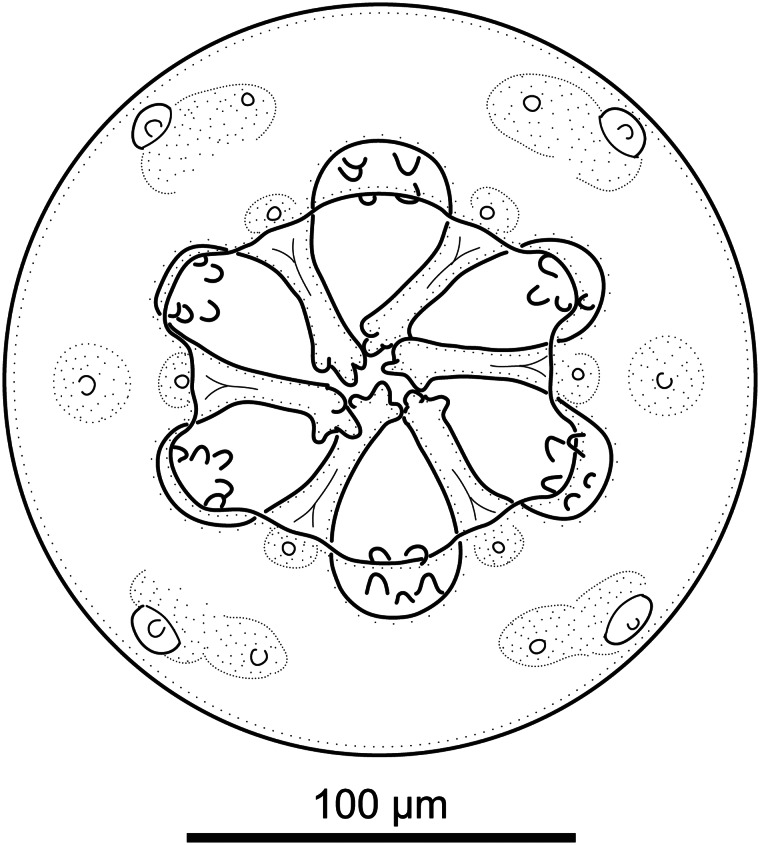
*Cylicospirura crocutae*. Female, apical view.

Of the 11 species of *Cylicospirura* described to date (Junker *et al*., 2013), only *C. subaequalis*, possesses bicuspid teeth (Vevers, 1922; Sandground, 1930; Waid and Pence, 1988). *Cylicospirura subaequalis* was described by Molin (1860) from the stomach of *Puma concolor* (Linnaeus) (as *Felis concolor* Linnaeus) and *Herpailurus yagouaroundi* (É. Geoffroy Saint-Hilaire) (as *Felis mellivora* Illiger) in Brazil. Unfortunately, the species description was not accompanied by illustrations and no reference was made to teeth in the buccal capsule. The most detailed description of *C. subaequalis* was that by Waid and Pence (1988) from *P. concolor* from Texas. The material from *H. hyaena* studied by us had originally been deposited in the MNHN as *Cylicospirura* sp., including the comment ‘non subaequalis’, indicating that the person studying these specimens did not consider them conspecific with *C. subaequalis*, despite the presence of bicuspid teeth. Similarly, both the specimens from *H. hyaena* and *Cr. crocuta* are distinct from *C. subaequalis* sensu Waid and Pence (1988). The most remarkable difference between the present specimens and *C. subaequalis*, is the shape of the bicuspid teeth in apical view. In their detailed drawings, Waid and Pence (1988; figs 5 and 10) illustrated the teeth of *C. subaequalis* as bicuspid, with a broad saddle-like space between the cusps; furthermore, the cusps are bevelled at the outer edge, giving each tooth a spanner-like appearance. In contrast, in the current specimens each of the 2 cusps is elongated with a rounded tip and with a deep incision between the 2 parallel cusps. The buccal capsule in the present specimens is distinctly longer than and roughly twice as wide as that of *C. subaequalis* (132–155 long by 133–160 wide *vs* 46–78 long by 59–82 wide in males, and 133–174 long by 185–204 wide *vs* 64–91 long by 73–96 wide in females). Moreover, the teeth project anteriorly beyond the mouth opening, while this is not the case in *C. subaequalis*. Waid and Pence (1988) emphasized that the vulva in females of *C. subaequalis* is always located posterior to the oesophago-intestinal junction, whereas in the present females its position is variable, either situated at the level of or anterior to the oesophago-intestinal junction and in some specimens posterior to it. Although males and females of the present specimens and those of *C. subaequalis* are of similar size, both the muscular and glandular oesophagus are shorter in *C. subaequalis* (133–215 *μ*m and 1200–1800 *μ*m, respectively, in males and 151–283 *μ*m and 1500–2600 *μ*m, respectively, in females). In addition, the left spicule is shorter in the present males when compared to that of *C. subaequalis* (2000–3300 *μ*m), whereas their right spicule is longer than that of *C. subaequalis* (313–402 *μ*m). The female tail is rounded in the species from hyaenas, whereas it is bluntly conical and bent ventrally in *C. subaequalis*. Based on the above-mentioned differences, we consider the present material from *Cr. crocuta* and *H. hyaena* distinct from *C. subaequalis* sensu Waid and Pence (1988) and belonging to an undescribed species for which we suggest the name *Cylicospirura phiri* Junker and Mutafchiev n. sp.

**Figure 6.**
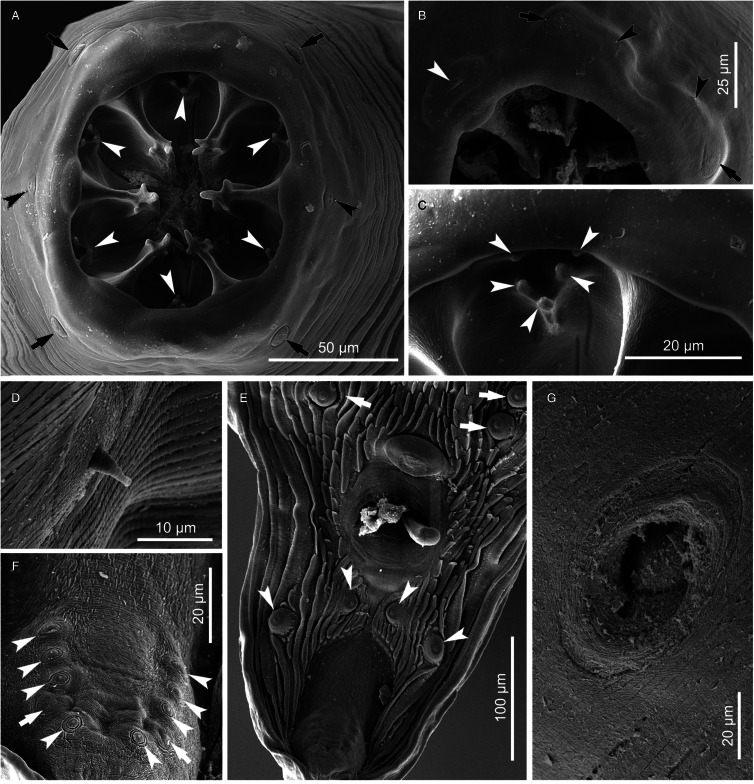
*Cylicospirura crocutae*, SEM. (A) Female, apical view, note cephalic papillae (arrows), amphids (black arrowheads) and accessory teeth (white arrowheads); (B) Detailed view of the anterior extremity of a female showing cephalic papillae (arrows), external labial papillae (black arrowheads) and an amphid (white arrowhead); (C) Accessory teeth (arrowheads), female. (D) Deirid, female; (E) Posterior extremity, male, note precloacal pedunculated papillae (arrows) and postcloacal pairs of pedunculated papillae (arrowheads); (F) Detail of the male caudal extremity, note sessile papillae (arrowheads) and phasmids (arrows); (G) vulva.

The current material resembles specimens identified as *C. subaequalis* and illustrated from *Panthera tigris* (Linnaeus) (as *Felis tigris* Linnaeus) from the Malaysia Peninsula by Vevers (1922; fig. 6), from ‘*Felis* sp.’ by Yorke and Maplestone (1926; fig. 202) and from *P. leo* in the Democratic Republic of the Congo by Sandground (1930). Moreover, all authors mention that the bicuspid teeth project slightly beyond the oral opening, at least in some specimens. Unfortunately, neither Yorke and Maplestone (1926) nor Vevers (1922) provide morphometric data of the specimens examined. However, measured on the caudal extremity of the male drawn by Vevers (1922), the spicular ratio is 1:12, *vs* a spicular ratio of 1:4.2–5.3 in the present males. Sandground's (1930) description of *C. subaequalis* was based on females only and the author emphasized the uncertainty surrounding specific determinations in the absence of comparative measurements. We cannot satisfactorily reconcile any of these reports to *C. phiri* n. sp. or *C. subaequalis* sensu Waid and Pence (1988).

#### *Cylicospirura crocutae* Junker and Mutafchiev, 2013

*Host*: *Crocuta crocuta* (Erxleben) (Carnivora: Hyaenidae); spotted hyaena.

*Locality*: Vicinity of Victoria Falls, Zimbabwe (17.vii.2017; -18.0170, 25.8359).

*Site in host*: Open granulomata in stomach.

*Prevalence and intensity of infection*: A single spotted hyaena harboured 22 specimens.

*Specimens deposited*: NCAH/2024/001 (3 males, 1 male anterior part, 4 females, 4 female anterior parts, 1 female posterior part). NHMW-ZOO-EV-A-21541 (1 male, 1 female, 2 female anterior parts), NHMW-ZOO-EV-M-5893 (1 female anterior end), NHMW-ZOO-EV-M-5894 (fragments of 1 male and 1 female).

#### Description (Figs 5 and 6; Tables 1 and 2)

*General*. For the full general description, the reader is referred to Junker *et al*. (2013). Body cylindrical, bent dorsally with maximum body width near oesophago-intestinal junction. Mouth opening hexagonal, slightly elongated laterally (Fig. 5). Large teeth 6, tricuspid, with small accessory teeth arranged in 6 groups of 4 to 6 between them (Figs 5 and 6A and C). Head papillae composed of an inner circle of 6 small internal labial papillae in lateral and submedian positions, close to mouth opening, visible using light but not electron microscopy, and an outer circle of 4 external labial papillae and 4 larger cephalic papillae, adjacent to each other and aligned with base of submedian large teeth (Figs 5 and 6A and B). Amphids aligned with lateral large teeth. Muscular oesophagus without pronounced anterior dilatation. Deirids small, spine-like, with flattened or bifid tip (Fig. 6D).

*Males* (based on 5 males, including 4 males and 1 anterior part; except where otherwise indicated). Body 22.2–26.0 (23.5; *n* = 4) mm long. Maximum width of body 460–646 (523). Buccal capsule 114–152 (142) long, including cylindrical base 38–61 (53) long and 46–80 (57) wide; maximum width of buccal capsule 138–161 (149). Deirids often asymmetrical, with anterior-most deirid at 435–530 (475) from anterior extremity and posterior-most deirid at 487–554 (523). Nerve-ring and excretory pore at 382–524 (466; *n* = 4) and 547–742 (663) from anterior extremity, respectively. Total length of oesophagus 5652–9615 (7179); i.e. 25.5–31.9% (28.0%; *n* = 4) of body length. Muscular oesophagus 696–987 (774) long and 112–130 (120) wide; glandular oesophagus 4938–8628 (6405) long and 225–302 (250) wide; ratio of muscular to glandular oesophagus length 1:6.9–9.0 (1:8.2). Testis extending to level slightly posterior to oesophago-intestinal junction (*n* = 1). Tail coiled ventrally, 310–355 (334; *n* = 4) long. Caudal alae narrow, 959–1367 (1163; *n* = 2) long. Area rugosa composed of distinct, parallel, longitudinal cuticular ridges; absent in area around cloaca and subterminal papillae; extending 2405–2750 (2551; *n* = 4) anteriorly from tip of tail. Cloaca slit-like, without conspicuous cuticular lips. Ten pairs of caudal papillae; pairs 1–4 more or less even-sized, ventrolateral, precloacal, pedunculated, not equidistant from each other, distance between pairs 2 and 3 larger than between pairs 1 and 2, and 3 and 4, respectively; pairs 5 and 6 postcloacal, pedunculated, pair 5 ventral, slightly smaller and slightly anterior to ventrolateral pair 6 (Fig. 6E); pairs 7–10 small, sessile, arranged in subterminal group around cuticular pads near tip of tail; phasmids situated between pairs 9 and 10 (Fig. 6F). One large, sessile, median papilla anterior to cloaca present (Fig. 6E). Spicules unequal and dissimilar; left spicule 2223–2554 (2358; *n* = 4) long and slender, tapering to pointed tip; right spicule 554–645 (597; *n* = 4) long, robust with round, hyaline distal tip; spicular ratio 1:3.7–4.1 (1:4.0; *n* = 4). Gubernaculum present, irregularly shaped, 80–90 (85; *n* = 4) long.

*Females* (based on 11 females, including 5 gravid females and 6 anterior parts of gravid females, except where otherwise indicated). Body 33.4–36.9 (35.0; *n* = 5) mm long. Maximum width of body 494–645 (611; *n* = 10); width at vulva 523–622 (571). Buccal capsule 112–190 (156) long, including cylindrical base 30–73 (51) long and 41–80 (57) wide; maximum width of buccal capsule 147–176 (168). Deirids often asymmetrical, with anterior-most deirid at 406–658 (523; *n* = 9) from anterior extremity and posterior-most deirid at 547–682 (605; *n* = 9). Nerve-ring and excretory pore at 458–588 (538; *n* = 9) and 586–829 (729; *n* = 9) from anterior extremity, respectively. Total length of oesophagus 6902–8849 (8310; *n* = 8); i.e. 19.8–25.0% (23.0%; *n* = 5) of body length. Muscular oesophagus 757–1095 (850; *n* = 10) long and 109–141 (127; *n* = 10) wide; glandular oesophagus 6055–8092 (7336; *n* = 9) long and 216–319 (272) wide; ratio of muscular to glandular oesophagus length 1:7.0–10.7 (1:8.8; *n* = 8). Uteri didelphic, opisthodelphic. Vulva without salient lips, opening at level of anterior half of glandular oesophagus, at approximately 26.0–43.3% (34.5%; *n* = 7) of glandular oesophagus length, and 9.8–11.2% (10.5%; *n* = 5) of body length; vagina simple, directed posteriorly, 700–1018 (890; *n* = 10) long; joined in its posterior third by ovejector that is convoluted and largely obscured by eggs in uterine loops. Eggs in uterus thin-shelled, oval, with well-developed first-stage larva; 34–37 (36; *n* = 11) × 18–23 (20; *n* = 11). Tail short, broadly conical, 90–149 (117; *n* = 6) long; tail length to width at anus ratio 1:1.7–2.2 (*n* = 3). Phasmids subterminal.

#### Remarks

The present specimens align well with the original description of *C. crocutae*. However, we report here the presence of 6 internal labial papillae, which in the specimens examined by Junker *et al*. (2013) had not been observed. Furthermore, we add morphometric data to the description of both males and females of this species, originally based on the holotype male and a single posterior male fragment, as well as 3 anterior and 2 posterior fragments of females. This provides a more complete picture of the range of morphological variation seen in *C. crocutae*.

#### *Cylicospirura pardalis* Junker and Mutafchiev, 2013

*Host*: *Panthera pardus* (Linnaeus) (Carnivora: Felidae); leopard.

*Locality*: Lower Sabie, Kruger National Park, South Africa (07.iv.2019; -25.12270, 31.91574)

*Site in host*: Granulomata in stomach (pyloric outlet), duodenum and jejunum.

*Intensity of infection*: A single leopard harboured 55 specimens.

*Specimens deposited*: NCAH/2024/002 (12 males, 5 females, 1 female posterior part). NHMW-ZOO-EV-A-21539 (2 males (1 partly torn), 1 female without anterior extremity), NHMW-ZOO-EV-M-5891 (1 female anterior extremity), NHMW-ZOO-EV-M-5892 (fragments of 1 male and 1 female).

#### Description (Figs 7 and 8, Tables 1 and 2)

**Figure 7.**
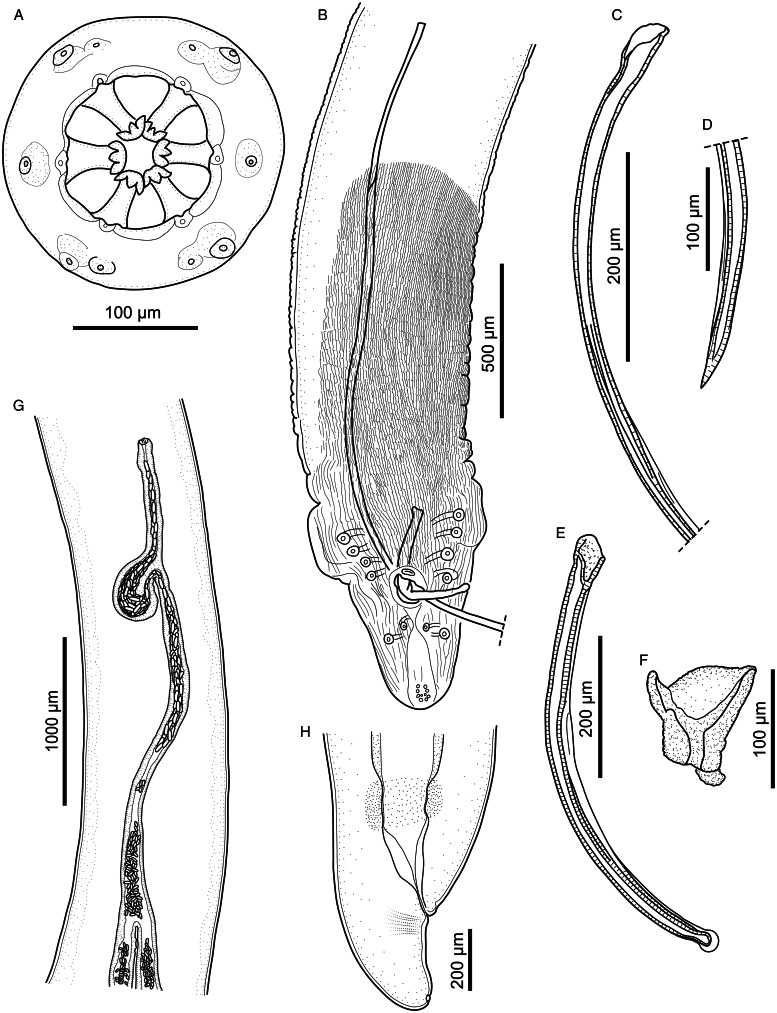
*Cylicospirura pardalis.* (A) Male, apical view; (B) Male, posterior end, ventral view; (C) Left spicule, proximal end; (D) Left spicule, distal end, lateral view. (E) Right spicule, lateral view; (F) Gubernaculum, dorsal view; (G) Terminal part of female genital system, ventral view; (H) Posterior end, female, lateral view.

**Figure 8.**
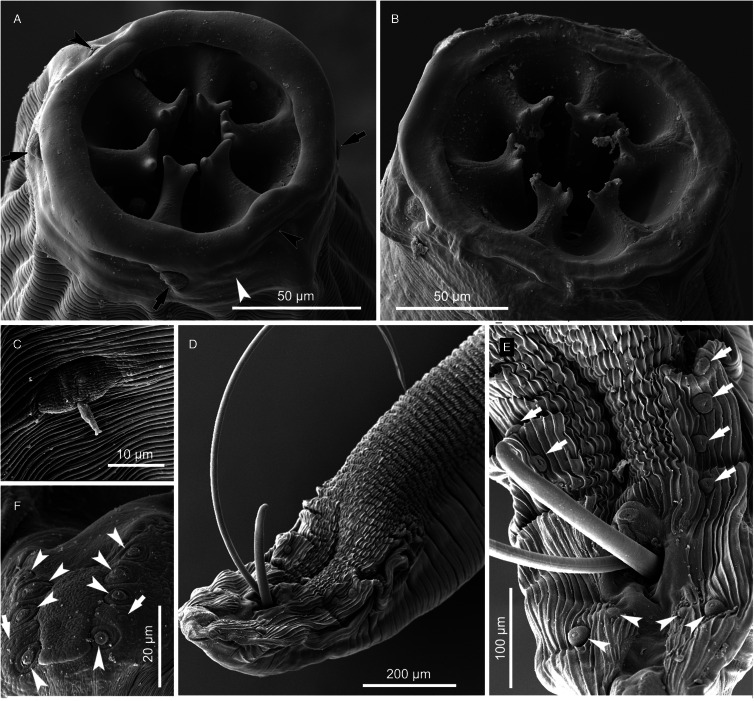
*Cylicospirura pardalis*, SEM. (A) Male, apical view, note cephalic papillae (arrows), amphids (black arrowheads) and a silent head papilla (white arrowhead); (B) Female, apical view; (C) Deirid, male; (D) Posterior end, male, ventrolateral view; (E) Cloacal region, note precloacal pedunculated papillae (arrows) and postcloacal pairs of pedunculated papillae (arrowheads); (F) Tail extremity, male, note the sessile papillae (arrowheads) and phasmids (arrows).

*General*. For the full general description, the reader is referred to Junker *et al*. (2013). Body cylindrical, bent dorsally with maximum body width near oesophago-intestinal junction. Large teeth 6, each tooth bearing 3 large, abaxial, claw-like cusps (Figs 7A and 8B); individual teeth may be bicuspid (Fig. 8A). Groups of small accessory teeth absent. Head papillae composed of an inner circle of 6 small internal labial papillae in lateral and submedian positions, close to mouth opening, visible using light but not electron microscopy, and an outer circle of 4 external labial papillae and 4 larger cephalic papillae, adjacent to each other and aligned with base of submedian large teeth. Amphids aligned with lateral large teeth (Figs 7A and 8A). Muscular oesophagus long and slender, a dilatation with valve close to its junction with buccal capsule. Deirids minute, spine-like, with bifid tip (Fig. 8C), at level of nerve ring or slightly anterior or posterior to it; excretory pore posterior to nerve ring and deirids, close to junction between muscular and glandular oesophagus; nerve ring, deirids and excretory pore at approximately mid-level of muscular oesophagus.

*Males* (based on 17 males; except where otherwise indicated). Body 18.5–32.2 (26.6) mm long. Maximum body width 485–641 (550), near oesophago-intestinal junction. Buccal capsule 125–187 (145) long, including cylindrical base 41–73 (57) long and 53–69 (61) wide; maximum width of buccal capsule 149–175 (160). Deirids often asymmetrical, with anterior-most deirid at 219–472 (405; *n* = 15) from anterior extremity and posterior-most deirid at 339–611 (461; *n* = 16). Nerve-ring and excretory pore at 332–533 (462; *n* = 16) and 398–671 (572; *n* = 15) from anterior extremity, respectively. Total length of oesophagus 3216–5549 (4400; *n* = 16); i.e. 14.7–19.6% (16.4%; *n* = 16) of body length. Muscular oesophagus 363–742 (612; *n* = 16) long and 106–138 (122) wide; glandular oesophagus 2720–4518 (3657; *n* = 16) long and 218–332 (266) wide; ratio of muscular to glandular oesophagus length 1:5.4–7.9 (6.2; *n* = 16). Reflexion of testis 2472–6260 (3827; *n* = 12) posterior to oesophago-intestinal junction. Tail coiled ventrally, 303–408 (361; *n* = 16) long. Caudal alae narrow, 898–962 (930; *n* = 2) long. Area rugosa composed of distinct, parallel, longitudinal cuticular ridges; absent in area around cloaca and subterminal papillae; extending 1727–4196 (3089; *n* = 14) anteriorly from tip of tail. Cloaca slit-like, without conspicuous cuticular lips. Ten pairs of caudal papillae; pairs 1–4 more or less even-sized, ventrolateral, precloacal and pedunculated, equidistant from each other, but in 1 specimen distance between pairs 2 and 3 larger than between pairs 1 and 2, and 3 and 4, respectively; pairs 5 and 6 postcloacal, pedunculated, pair 5 ventral, slightly smaller and slightly anterior to ventrolateral pair 6 (Figs 7B and 8E); pairs 7–10 small, sessile, arranged in subterminal group around cuticular pads near tip of tail; phasmids situated between pairs 9 and 10 (Fig. 8F). A single, large, sessile, median papilla anterior to cloaca. Spicules unequal and dissimilar; left spicule 2485–2860 (2647; *n* = 16) long and slender, tapering to pointed tip; right spicule 517–701 (595; *n* = 16) long, robust with round, hyaline distal tip (Figs 7C–E and 8D); spicular ratio 1:3.9–4.9 (1:4.5; *n* = 16). Gubernaculum present, irregularly shaped, 86–115 (98; *n* = 13) long (Fig. 7F).

*Females* (based on 8 gravid females; except where otherwise indicated). Body 29.0–45.5 (38.0) mm long. Maximum body width at vulva 674–861 (783); body width at oesophago-intestinal junction 674–827 (756; *n* = 7). Buccal capsule 150–223 (183) long, including cylindrical base 56–79 (68) long and 69–84 (76) wide; maximum width of buccal capsule 186–240 (216). Deirids often asymmetrical, with anterior-most deirid at 423–578 (515; *n* = 7) from anterior extremity and posterior-most deirid at 491–658 (588; *n* = 7). Nerve-ring and excretory pore at 490–629 (566; *n* = 6) and 670–815 (731; *n* = 6) from anterior extremity, respectively. Total length of oesophagus 4576–5753 (5210; *n* = 7); i.e. 12.0–14.4% (13.3%; *n* = 7) of body length. Muscular oesophagus 665–869 (740; *n* = 7) long and 133–187 (154) wide; glandular oesophagus 3872–4994 (4470; *n* = 7) long and 288–364 (329; *n* = 7) wide; ratio of muscular to glandular oesophagus length 1:5.1–6.6 (1:6.1; *n* = 7). Uteri didelphic, opisthodelphic. Vulva without salient lips, opening 4113–5512 (4677; *n* = 7) from anterior extremity, at level of posterior half of glandular oesophagus, at approximately 76.4–91.4% (84.0%; *n* = 7) of glandular oesophagus length, and 10.7–13.5% (11.9%; *n* = 7) of body length; vagina simple, directed posteriorly, 955–1102 (1029; *n* = 10) long; joined in its posterior third by ovejector that is convoluted and largely obscured by eggs in uterine loops; vagina and ovejector 1097 and 2275 long, respectively, in single anterior fragment measured (Fig. 7G). Eggs in uterus thin-shelled with almost parallel sides and with well-developed first-stage larva; 36–38 (37; *n* = 8) × 14–17 (16; *n* = 8). Tail conical (Fig. 7H), 264–334 (289) long; tail length to width at anus ratio 1:0.9–1.1 (*n* = 3). Phasmids subterminal.

#### Remarks

The morphological features of the current specimens are consistent with the description of *C. pardalis* by Junker *et al*. (2013). While clearly distinct from its congeners by the morphology of its teeth, several morphological characters that could not be observed in the original specimens, which comprised one male anterior and posterior fragment as well as on one female anterior fragment, have here been added to the description. Contrary to the original specimens, in which external labial papillae could not be seen, these are present in the current specimens and group closely with the cephalic papillae (Fig. 7A). Body size of males and females could be determined for the first time, indicating that *C. pardalis* is one of the largest *Cylicospirura* species in African carnivores (Tables 1 and 2). Similarly, first time data are presented for the dimensions of the buccal capsule in males, the arrangement of postcloacal papillae and length of the left spicule.

#### *Cylicospirura felinea* (Chandler, 1925) Sandground, 1933

*Host*: *Felis lybica* Forster (Carnivora: Felidae); African wildcat.

*Locality*: Algeria; collection date not specified.

*Site in host*: Not specified.

*Prevalence and intensity of infection*: Not specified.

*Specimens examined*: MNHN IN YT113; originally deposited as ‘*Cylicospirura subaequalis’* from ‘*Felis ocreata’*.

#### Description (Figs 9 and 10, Tables 1 and 2)

**Figure 9.**
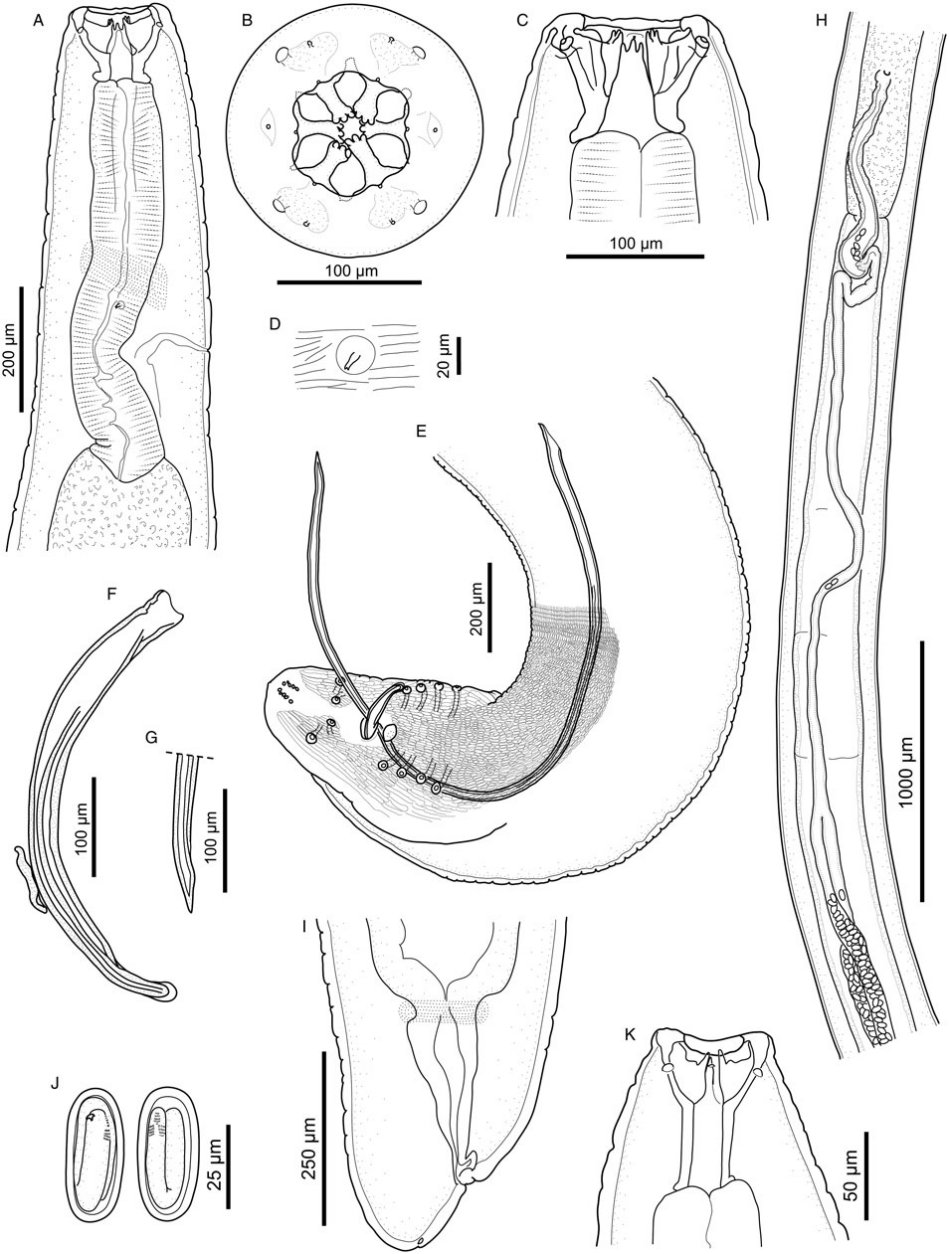
*Cylicospirura felinea.* (A) Anterior end, female, lateral view; (B) Female, apical view; (C) Anterior extremity, male, lateral view; (D) Deirid; (E) Male, posterior end, semi ventral view; (F) Right spicule and gubernaculum, dextral view; (G) Distal end of left spicule; (H) Vagina, ovejector and distal part of uterus; (I) Posterior end, female, lateral view; (J) Eggs; (K) Anterior extremity, fourth-stage larva, lateral view.

**Figure 10.**
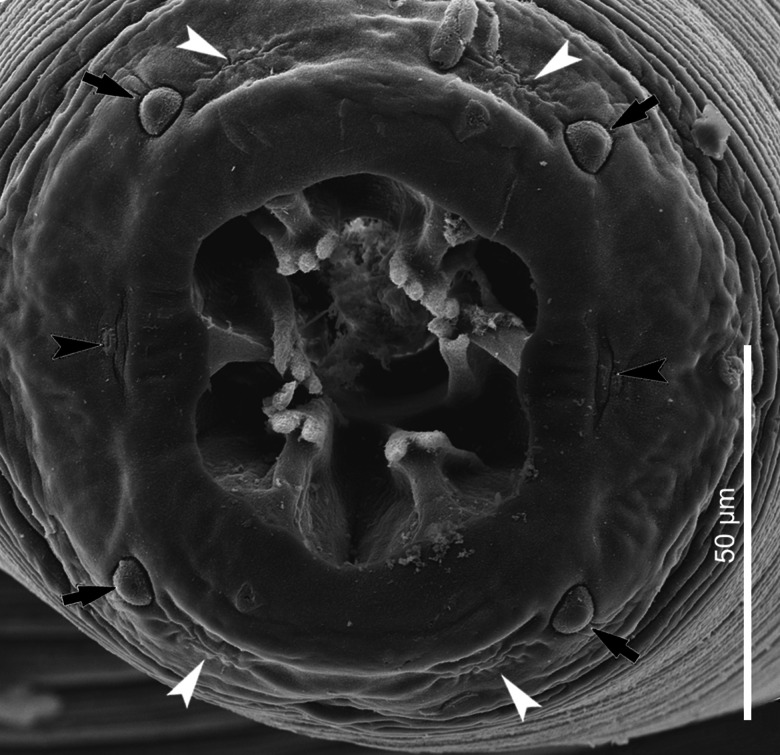
*Cylicospirura felinea*, SEM. Female, apical view, note cephalic papillae (arrows), amphids (black arrowheads) and position of the external labial papillae (white arrowheads).

*General*. Body bent dorsally and tapering at both ends, with maximum body width at level of oesophago-intestinal junction. Cuticle with narrow transverse striations, spaced approximately 3 *μ*m apart. Lateral alae absent. Mouth opening hexagonal (Figs 9B and 10). Buccal capsule large, heavily sclerotized, bowl-shaped, with squat cylindrical base representing approximately 40% of its length (Fig. 9C). Six large cuticular teeth arising in posterior half of buccal capsule, projecting anteriorly; each tooth tricuspid, with well-developed, symmetrical, rounded cusps (Figs 9B and 10). Groups of small accessory teeth absent. Head papillae composed of an inner circle of 6 small internal labial papillae in lateral and submedian positions, close to mouth opening, visible using light but not electron microscopy, and an outer circle of 4 external labial papillae and 4 larger cephalic papillae, adjacent to each other and aligned with base of submedian large teeth. Amphids aligned with lateral large teeth (Figs 9B and 10). Oesophagus divided into anterior muscular and posterior glandular part; muscular oesophagus long and slender, nearly cylindrical; anterior part of glandular oesophagus distinctly wider than posterior part of muscular oesophagus (Fig. 9A); glandular oesophagus cylindrical. Deirids small, bifid (Fig. 9D), at level of nerve ring or slightly anterior or posterior to it; excretory pore posterior to nerve ring and deirids; nerve ring at approximately mid-level of muscular oesophagus (Fig. 9A and D).

*Males* (based on 1 entire male, 2 anterior and 3 posterior parts; number of measurements indicated in parentheses). Body 20.8 (*n* = 1) mm long. Maximum body width 433–466 (*n* = 3). Buccal capsule 93–102 (*n* = 3) long, including cylindrical base 31–40 (*n* = 3) long and 73–80 (*n* = 2) wide; maximum width of buccal capsule 134–145 (*n* = 3). Nerve-ring and deirids at 316–377 (*n* = 2) and 309–391 (*n* = 3) from anterior extremity, respectively. Excretory pore at 410–453 (*n* = 2) from anterior extremity. Total length of oesophagus 3959–4061 (*n* = 2); i.e. 19.5% (*n* = 1) of body length. Muscular oesophagus 474–564 (*n* = 2) long and 106–130 (*n* = 3) wide; glandular oesophagus 3485–3497 (*n* = 2) long and 243–286 (*n* = 2) wide; ratio of muscular to glandular oesophagus length 1:6.2–7.4 (*n* = 2). Posterior end coiled ventrally, tail 218–246 (*n* = 3) long. Caudal alae broad. Area rugosa composed of distinct, parallel, longitudinal cuticular ridges; absent in area around cloaca and subterminal papillae (Fig. 9E). Cloaca slit-like, without conspicuous cuticular lips. Ten pairs of caudal papillae; precloacal pairs 1–4 more or less even-sized, ventrolateral, pedunculated, approximately equidistant from each other; pairs 5 and 6 postcloacal, pedunculated, pair 5 ventral, slightly smaller and slightly anterior to ventrolateral pair 6; pairs 7–10 small, sessile, arranged in subterminal group around cuticular pads near tip of tail; phasmids situated between pairs 9 and 10. A single, large, sessile, median papilla anterior to cloaca. Spicules unequal and dissimilar; left spicule 1866–2038 (*n* = 4) long and slender, tapering to pointed tip (Fig. 9E and G); right spicule 423–472 (*n* = 3) long, robust with round, hyaline distal tip (Fig. 9F); spicular ratio 1:4.0–4.4 (*n* = 2). Gubernaculum present, not clearly observed.

*Females* (based on 3 entire females and 1 female anterior part; except where otherwise indicated). Body 16.7–21.2 (*n* = 3) mm long. Maximum body width in posterior third of body, 558–693 (*n* = 3); width at oesophago-intestinal junction 357–505; width at vulva 371–484. Buccal capsule 109–126 long, including cylindrical base 41–46 long and 73–98 wide; maximum width of buccal capsule 140–160. Nerve-ring and deirids at 362–430 and 296–509 from anterior extremity, respectively. Excretory pore at 422–523 (*n* = 3) from anterior extremity. Total length of oesophagus 4119–5125; i.e. 22.3–29.7% (*n* = 3) of body length. Muscular oesophagus 509–620 long and 105–123 wide; glandular oesophagus 3610–4506 long and 206–284 wide; ratio of muscular to glandular oesophagus length 1:6.6–7.3. Uteri didelphic, opisthodelphic. Vulva without salient lips, opening 3045–4265 from anterior extremity, at level of posterior half of glandular oesophagus, 551–1663 anterior to oesophago-intestinal junction, at approximately 62.6–86.1% of glandular oesophagus length, and 19.8–22.8% (*n* = 3) of body length; vagina simple, directed posteriorly, 625–846 (*n* = 2) long; joined in its posterior third by ovejector that is convoluted and largely obscured by eggs in uterine loops (9H). Eggs in uterus thick-shelled, oval and with well-developed first-stage larva (Fig. 9J); 39–40 × 20–22 (*n* = 4). Tail broadly conical (Fig. 9I), 96–134 (*n* = 3) long; tail length to width at anus ratio 1:1.4–1.8 (*n* = 3). Phasmids subterminal.

*Fourth-stage larva* (based on 1 specimen). Body 8.8 mm long and 277 wide. Buccal capsule 80 long and 62 wide, with elongated cylindrical base; armed with 6 large cuticular teeth, each with single pointed tip (Fig. 9K). Muscular oesophagus 336 long and 68  wide. Glandular oesophagus 2386 long and 148 wide. Deirid 205, nerve ring 210 and excretory pore 277 from anterior extremity. Vulva at 1688 from anterior extremity. Body width at anus 79. Tail length 30. Tail length to width at anus ratio 1:2.6.

#### Remarks

The specimens from *F. lybica* from Algeria conform well to the description of specimens erroneously recorded by Seurat (1913) as ‘*Spiroptera subaequalis* Molin’ from a ‘chat ganté (*Felis ocreata* Gmel.)’ in Algeria, that of *C. felinea* (as *Spirocerca felinea*) from domestic cats in India (Chandler, 1925), from *Prionailurus bengalensis* (Kerr) (as *Felis bengalensis* Kerr) in Vietnam (Sandground, 1933), as well as that of *C. felinea* from *Lynx canadensis* Kerr (as *Felis canadensis* (Kerr)) and *Lynx rufus* (Schreber) (as *Felis rufus* (Schreber)) in North America (Pence *et al*., 1978). Despite having similar collection details (deposited as ‘*Cylicospirura subaequalis’* from ‘*Felis ocreata’*), it was no longer possible to confirm if the present specimens belonged to those originally examined by Seurat (1913). The present specimens and those described by previous authors are characterized by having tricuspid teeth, with finger-like cusps in lateral view (Seurat, 1913; Chandler, 1925) and well-developed, symmetrical rounded cusps in apical view (Seurat, 1913; Sandground, 1933; Pence *et al*., 1978). Concerning the morphology of their teeth, they also conform to specimens of *C. felinea* from a domestic cat in South Africa (Junker *et al*., 2006). It is noteworthy that Chandler (1925, fig. 4) commented that the mouthparts (tricuspid teeth) of *C. felinea* were usually withdrawn into the buccal capsule but could be extruded under pressure.

Both the head and caudal papillae can be difficult to observe in *Cylicospirura* spp. and different authors recorded varying numbers of these papillae for *C. felinea*. Seurat (1913) neither commented on the number of head nor caudal papillae, whereas Chandler (1925) stated that cephalic papillae were absent and caudal papillae consisted of 4 precloacal and 1 postcloacal pair. Sandground (1933) illustrated 4 cephalic papillae as well as 4 precloacal and 2 postcloacal pairs of caudal papillae. In addition, he illustrated what can be interpreted as the group of 4 pairs of subterminal papillae but did not refer to them as such. Pence *et al*. (1978) identified 6 small papillae between adjacent crescents encircling the mouth opening and 4 large cephalic papillae in *C. felinea* from North American *Lynx* spp., similar to the arrangement of head papillae in the present specimens. However, in the specimens from *F. lybica* we were also able to identify 4 external labial papillae. These external labial papillae are usually less distinct than the cephalic ones and arranged in close proximity to them, as seen for example in *C. crocutae*, *C. pardalis* and *C. phiri* n. sp. Pence *et al*. (1978) were the first to document the complete set of caudal papillae in *C. felinea*, including the subterminal group of 4 pairs of sessile papillae, with the phasmids situated between the last 2 pairs, and the single median papilla in front of the cloaca.

## Discussion

Based on previous (Junker *et al*., 2013) and present findings, the morphology of the large teeth as well as the absence or presence of groups of small accessory teeth remain the most reliable characters to distinguish between *Cylicospirura* species. In African carnivores, *C. phiri* n. sp. is to date the only species with bifid teeth, whereas *C. crocutae* is the only species with groups of small accessory teeth between the large teeth. Unfortunately, it is not always possible to determine tooth morphology in lateral or dorsoventral view. An apical view allows for a more reliable confirmation of the number of cusps and their configuration but brings with it the necessity of destructive sampling.

It might thus be helpful to consider qualitative morphological characters (Table 3) in addition to the morphometrics listed in Tables 1 and 2, when studying *Cylicospirura* spp. from African carnivores. *Cylicospirura phiri* n. sp. is the only *Cylicospirura* species with conspicuous transverse rugosities marking the buccal capsule, and the base of the capsule is distinctly narrower compared to that of the remaining 3 species. Considering the shape of the muscular oesophagus, that of *C. phiri* n. sp. appears short and stout, when compared to that of its 3 congeners, in which it is long and slender. While we did not observe a pronounced dilatation in the anterior region of the muscular oesophagus in *C. felinea* or *C. crocutae*, it was present in *C. pardalis* and *C. phiri* n. sp. The nerve-ring, deirids and excretory pore were positioned in the midregion of the muscular oesophagus in *C. crocutae*, *C. pardalis* and *C. felinea*, but at a distinctly posterior level of the muscular oesophagus in *C. phiri* n. sp.

**Table 3.** Morphological characters of *Cylicospirura* spp. from African carnivores

Species	*C. crocutae*	*C. pardalis*	*C. phiri*	*C. felinea*
Morphology of teeth	Trisucpid: lateral cusps rounded, knob-like, central cusp slightly elongated with rounded tip	Tricuspid: large, abaxial cusps claw-like. Some teeth maybe bicuspid	Bicuspid: each cusp elongated with rounded tip and with deep split between 2 cusps	Tricuspid: well-developed, symmetrical rounded cusps
Accessory teeth	Present	Absent	Absent	Absent
Shape of muscular oesophagus	Long, slender, no pronounced anterior dilatation	Long, slender, with pronounced anterior dilatation	Short, stout, with pronounced anterior dilatation	Long, slender, no pronounced anterior dilatation
Level at which nerve ring is situated	Mid-region of muscular oesophagus	Mid-region of muscular oesophagus	Posterior region of muscular oesophagus	Mid-region of muscular oesophagus
Position of vulva in relation to GIT	Anterior half of glandular oesophagus	Posterior fourth of glandular oesophagus	Post half of gland oesophagus or posterior to oesophago-intestinal junction	Posterior half of glandular oesophagus
Tail shape in females	Short, broadly conical	Conical, bend ventrally with rounded tip	Rounded	Short, broadly conical
Eggs	Thin-shelled	Thin-shelled	Thin-shelled	Thick-shelled

*Cylicospirura crocutae* is the only species in which the vulva is positioned at the anterior half of the glandular oesophagus (Table 2). In the remaining species, it is situated at the posterior half of the oesophagus or, as in some specimens of *C. phiri* n. sp., posterior to the oesophago-intestinal junction. Lastly, the tail is short and broadly conical in females of *C. crocutae* and *C. felinea*, but somewhat more elongated in *C. pardalis* and rounded in *C. phiri* n. sp. The disposition of the 4 pairs of precloacal pedunculated papillae was equidistant in *C. phiri* n. sp. and split into an anterior and posterior group of 2 pairs each in *C. crocutae*. The disposition of these papillae in *C. pardalis* was somewhat intermediate, not equidistant, but also not clearly divided into 2 groups, and might have been influenced by muscle contraction. In a single male of *C. felinea* in which the precloacal papillae could be studied, they were evenly spaced.

Based on light microscopy studies of the apical view, we identified the presence of 2 lateral amphids, 4 large cephalic, 4 smaller external labial and 6 internal labial papillae in *C. crocutae*, *C. phiri* n. sp., *C. pardalis* and *C. felinea*. The cephalic and external labial papillae are arranged adjacent to each other on an outer circle approximately on the level of the amphids or slightly posterior, while an inner circle contains the internal labial papillae close to the mouth opening. Interestingly, only the amphids and cephalic papillae could be readily confirmed on the SEM micrographs of these species. External labial papillae were seen on SEM micrographs of *C. crocutae* and *C. felinea*, but not of *C. phiri* n. sp. and *C. pardalis*. Internal labial papillae could not be confirmed *via* SEM for any of the 4 species. In the closely related spirocercines *Spirocerca vulpis* Rojas *et al*., 2018 and *Spirocerca lupi* (Rudolphi, 1819), Rojas *et al*. (2018; fig. 4B) and Hoa *et al*. (2021; fig. 1A), respectively, used SEM to demonstrate 4 submedian cephalic papillae and 2 lateral amphids. However, a close look at these SEM micrographs reveals the presence of a pore-like opening in the cuticle surface next to the raised cushion of the cephalic papilla on its median side. Similarly, Quentin (1973), when studying *Spirura guianensis* (Ortlepp, 1924) (Spiruridae), described 2 amphids, 4 cephalic papillae, and 4 external labial papillae, the latter hidden under the buccal cadre. SEM studies of *S. guianensis* by Torres *et al*. (2015) only revealed the amphids and cephalic papillae. Based on light microscopy, Pence *et al*. (1978; fig. 16) identified 4 cephalic, 4 external labial and 6 internal labial papillae in *Cyathospirura chevreuxi* (Seurat, 1913), another closely related spirocercine. This suggests that 4 large cephalic, 4 smaller external labial and 6 internal labial papillae likely represent the ancestral complement of head papillae in these spirocercines. However, depending on the state of preservation of specimens or muscular contraction of the anterior end, detection of the more delicate pore-like external labial papillae can be difficult.

While the internal labial papillae remained obscure on all SEM images, confirmation of internal labial papillae in *C. crocutae via* light microscopy, suggests that this is a common feature within the genus, recorded for 8 of the now 12 species (Kozlov *et al*., 1964; Mawson, 1968; Pence *et al*., 1978; Clark, 1981; Waid and Pence, 1988; Junker *et al*., 2013; this paper); no information on the presence or absence of internal labial papillae is available for *C. arctica* (Petrow, 1927), *C. barusi* (Arya, 1979), *C. petrowi* (Sadykhov, 1957) and *C. lyncis* (Matschulsky, 1952) (see Petrow, 1927; Matschulsky, 1952; Sadykhov, 1957; Arya, 1979).

The fourth-stage larva described from *F. lybica* in Algeria differs from adults in the sample identified as *C. felinea* in having a narrower buccal capsule armed with teeth with a single cusp instead of 3 cusps. It is worth mentioning that the buccal capsule of this fourth-stage larva is morphologically similar to that of *C. arctica* from *Vulpes lagopus* (Linnaeus) as illustrated by Petrow (1927), which may suggest that the author in fact documented an advanced fourth-stage female instead of an adult female. Future studies should thus pay attention to possible discrepancies between the original description of *C. arctica* and new materials collected from Arctic carnivores.

Of the 4 *Cylicospirura* species in African carnivores, *C. crocutae* from *Cr. crocuta* and *C. pardalis* from *P. pardus*, have to date been recorded from a single host species only (Junker *et al*., 2013; this paper), suggesting a limited host as well as geographic range. However, this view might be skewed by the scarcity of data on the parasites of African carnivores in general. In contrast, *C. felinea* has been reported from several felid (Felinae and Pantherinae), but also canid hosts (Junker *et al*., 2013), and the description of *C. phiri* n. sp. from *H. hyaena* and *Cr. crocuta* (Hyaenidae) in Central and southern Africa, respectively, emphasizes the adaptability of *Cyclicospirura* species to different hosts and varying environmental conditions.

Our finding of *C. crocutae* and *C. phiri* n. sp. in *Cr. crocuta* from Zimbabwe constitutes the first report of a mixed infection with more than 1 species of *Cylicospirura*. Interestingly, *C. crocutae* was found in open granulomata in the stomach, while *C. phiri* n. sp. occurred free in the cranial part of the oesophagus, excepting 2 males collected from the open granulomata in the stomach. More data are needed to confirm whether this is a common pattern of infection or a random finding.

## Data Availability

All data generated or analysed during this study are included in this published article. The datasets used and/or analyses are available from the corresponding author upon reasonable request.
